# Developing a New Spectral Index for Detecting Cadmium-Induced Stress in Rice on a Regional Scale

**DOI:** 10.3390/ijerph16234811

**Published:** 2019-11-29

**Authors:** Chuanyu Wu, Meiling Liu, Xiangnan Liu, Tiejun Wang, Lingyue Wang

**Affiliations:** 1 School of Information Engineering, China University of Geosciences, Beijing 100083, China; 2 Faculty of Geo-Information Science and Earth Observation (ITC), University of Twente, 7500 Enschede, The Netherlands

**Keywords:** cadmium stress, sensitive spectral index, radiative transfer model, regional scale, sentinel-2A image

## Abstract

In natural farmland ecosystems, cadmium (Cd) pollution in rice has attracted increasing attention because of its high toxicity, relative mobility, and high water solubility. This study aims to develop a spectral index for detecting Cd stress in rice on a regional scale. Three experimental sites are selected in Zhuzhou City, Hunan Province. The hyperspectral data, chlorophyll (Chl) content, leaf area index, average leaf angle, Cd concentration in soil, and Sentinel-2A images from 2017 and 2018 are collected. A new spectral index sensitive to Cd stress in rice is established based on the global sensitivity analysis of the radiative transfer model PROSPECT + SAIL (commonly called PROSAIL) model with the auxiliary of the field-measured data. The heavy metal Cd stress-sensitive spectral index (HCSI) is devised as an indicator of the degree of Cd stress in rice. Results indicate that (1) the HCSI developed based on Chl is a good indicator of rice damage caused by Cd stress, that is, low values of HCSI occur in rice subject to relatively high pollution; (2) compared with common spectral indices, such as red-edge position and red-edge Chl index, HCSI is more sensitive to Chl content with higher Pearson correlation coefficients with respect to Chl content, ranging from 0.85 to 0.95; (3) HCSI is successfully applied in Sentinel-2A images from the two different years of monitoring rice Cd stress on a regional scale. Cd stress levels in rice stabilized, and the largest area percentage of each pollution levels of Cd decreased in the following order: No pollution (i.e., 40%), low pollution (i.e., 35%), and high pollution (i.e., 25%). This study indicates that a combination of simulation data from the PROSAIL model and measured data appears to be a promising method for establishing a sensitivity spectral index to heavy metal stress, which can accurately detect regional Cd stress in crops.

## 1. Introduction

Heavy metal stress is defined as any heavy metal pollutant that is potentially unfavorable to crops’ metabolism, growth, or development [1]. With the development of industry, some heavy metal pollutants have caused farmland disturbance in developing countries, typically cadmium (Cd), copper (Cu), zinc (Zn), lead (Pb), and others [2]. Cd stress is potentially unfavorable to rice metabolism, growth, or development, which is one of the critical food pollution problems in China [3]. The adsorption of Cd in rice is higher than that of other pollutants; thus, Cd pollution in rice is relatively obvious [4]. High concentrations of Cd pollution can have a negative effect on vegetation [5]. Cd is absorbed by rice, causing rice pollution, which in turn harms the environment and the health of animals and plants [6]. Therefore, detecting Cd pollution plays a crucial role in assessing crop quality, environmental quality, and human health. 

Heavy metal pollution can lead to a change in vegetation color and morphogenesis, leaf yellowing, vegetation branching, defoliation, and even death [7,8,9,10,11]. Studies have shown that Cd stress has a similar effect. Cd stress causes a reduction in rice growth; excessive Cd in the growth reduces rice seed germination, causea leaf chlorosis and necrosis; and photosynthesis decreases in rice, which further results in decreasing chlorophyll (Chl), leaf area index (LAI), and changes in average leaf angle (ALA) [12,13,14]. Such pollution can also destroy the internal structure of vegetation leaves. Zhu et al. [15] found that with an increase in copper content, the mesophyll cells shrink, the internal structure of leaves appears disordered, and the leaf structure parameter (N) increases. The spectral reflectance of vegetation changes considerably because of the remarkable changes in the various biochemical and biophysical parameters of vegetation.

Traditionally, assessment of Cd stress has often been conducted through soil testing, crop tissue analysis, and long-term field trials in sequential steps with increasing high cost. Remote sensing technology provides an efficient and economical means for assessing crop Cd stress over large areas [16]. Many studies have shown that remote sensing has been used for monitoring crops in controlled laboratory conditions or specific field environments under heavy metal stress [9,17,18]. Vegetation spectral indices are among the common methods that can characterize the variation in vegetation in response to heavy metal stress [19]. For example, spectral indices, such as normalized difference vegetation index, modified chlorophyll absorption ratio index, structure insensitive pigment index, enhanced vegetation index, red-edge chlorophyll index (Clre), red-edge position (REP), and normalized red-edge differences (NDRE), are commonly used to detect crops under heavy metal stress [20,21,22,23,24]. Kooistra et al. [25] used REP and Cd in soil for regression analysis, and found that REP had a good correlation with Cd content. Likewise, many spectral signal enhancement methods, such as principal component regression, independent component analysis, wavelet transform, and spectral absorption depth, have been used to extract the spectral characteristics of stressed vegetation [26,27,28]. Many studies have also applied partial least square regression, multiple linear regression, and artificial neural network methods to establish an empirical relationship between spectral reflectance data and vegetation biochemical and biophysical parameters; this relationship is used to identify heavy metal stress in vegetation [19,29,30,31,32]. Liu et al. [33] used a back propagation neural-network model to estimate chlorophyll concentration in rice under Cd stress.

Heavy metal stress under laboratory conditions or specific field-scale environments (e.g., nearby abandoned mines) can cause considerable changes in vegetation, such as obvious leaf yellowing and leaf curls [12,13,34]. Thus, different stress levels in vegetation are easy to detect. Spectral indices that are sensitive to vegetation biochemical and biophysical parameters have been established on the basis of substantial field measurement data in experiments under heavy metal pollution in laborious or field scale. These field-scale approaches lack universality and cannot be used for large-scale information monitoring [27,32]. Detecting the level of pollutants in “real-world” ecosystems is relatively difficult, and how to use biochemical parameters, biophysical parameters, or spectral wavelengths in vegetation in response to heavy metal remains less explored. Li et al. [35] constructed the hyperspectral index based on subtle changes in canopy chlorophyll, and used Hyperion images to monitor canopy chlorophyll under As stress on a large scale. Liu et al. [36] integrated physiological function variability with spatio-temporal stability using multi-temporal thermal remote sensing for regional monitoring of heavy metal stress. To further assess large-area heavy metal concentrations in crops, the establishment of new vegetation spectral indices is needed based on the selection of sensitive wavelengths by integrating measurement data with simulation data.

Vegetation radiative transfer models (RTMs) have recently been widely used for vegetation parameter detection [37,38,39,40], and many people have used vegetation RTMs to develop spectral indices. Zhou et al. [41] used the PROSPECT-5 model to establish a new spectral index for estimating the ratio of carotenoids to Chl content to monitor the physiological and phenological conditions of crops. Pasqualotto et al. [42] utilized the PROSAIL model constructs with two spectral indices that accurately estimate the water content of the canopy. Among many available vegetation radiative transfer models, this study selected the PROSAIL model, which is coupled by leaf and canopy radiative transfer model. The model was chosen because of its availability, accuracy, and simplicity [39]. The PROSAIL model is a physical model that provides a thorough understanding of biological and biochemical processes, analyzes signals from remote sensing data, allows input, and generates rich simulation datasets [40]. The PROSAIL can not only simulate the spectral characteristics of different crop types under different physiological conditions, but can also compensate for the deficiency of measured data in field experiments (e.g., insufficient data, lack of key data, uncontrollable environmental parameters, and unrepeatable experiments) [39,43,44]. In addition, spectral wavelengths that are sensitive to specific biochemical and biophysical parameters are determined, and spectral indices that are sensitive to different pollution types in farmland can be explored. The spectral indices developed by PROSAIL model have good universality and can be used on a large scale in similar crop type research [39].

Satellite remote sensing images provide an efficient, economical, and large-scale monitoring method to verify the application of a spectral index developed by vegetation RTMs and monitor Cd stress in farmland on a large scale. Xiao et al. [45] developed NDII as the land surface water index based on the SPOT-4 satellite image and used it to estimate water stress coefficient. Muramatsu et al. [46] constructed a new index and applied it to Landsat satellite images for land-cover classification. Sentinel-2 satellite remote sensing images have a wide application in distinguishing different crop types, monitoring plant stress, and retrieving biophysical parameters, such as LAI, leaf Chl content, and leaf water content (Cw) [47,48,49,50,51,52]. They contain three red-edge bands that provide key information for monitoring the vegetation state. Several studies have confirmed that when plants are subjected to heavy metal stress, the red-edge parameters of plant spectra change [25,53,54]. Therefore, Sentinel-2 can obtain more information about plant stress changes than SPOT and Landsat satellite images.

This study focuses on the development of a spectral index that can accurately monitor different Cd stress levels in rice on a large scale. The contributions of this study are as follows: (1) A new Cd stress-sensitive spectral index is established using the PROSAIL model, which can explore the sensitive wavelength bands and biochemical components of rice in response to Cd stress; (2) the common spectral indices and the newly developed spectral index are compared using field-measured and simulated data; (3) the newly developed spectral index is upscaled to Sentinel-2 remote sensing images to detect Cd stress levels in rice on a large scale.

## 2. Study Area and Data

### 2.1. Study Area

**Figure 1:**
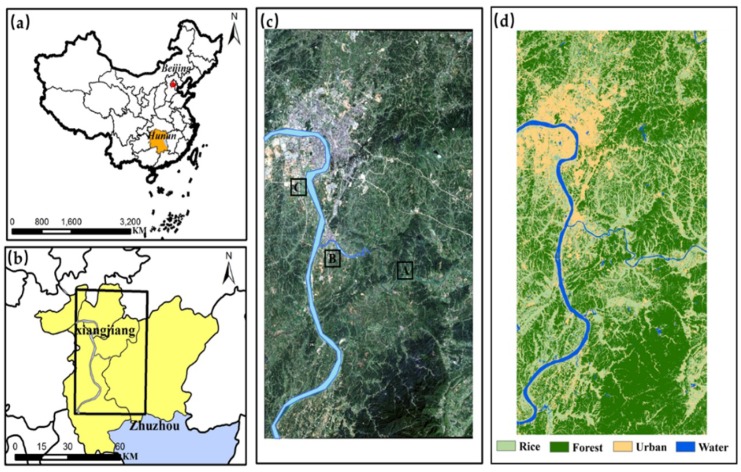
Location of the study area: (**a**) General location in China; (**b**) location in the north of Zhuzhou, Hunan Province; (**c**) Sentinel-2 true-color image of the study area; (**d**) land use classification of study area.

The study area is located in Zhuzhou, Hunan Province, China (Figure 1), which is a famous commodity grain production region with 230,000 tons of rice per year and an important industry in China. It is located in northeastern Hunan Province and the lower reaches of the Xiangjiang River (112°57′30″–114°07′15″ E, 26°03′05″–28°01′07″ N). The climate of the region is subtropical monsoon, with an average annual temperature of 16–18 °C. The predominant soil type is red soil with sufficient nutrient content. Mountain areas are mainly concentrated in the southeast of the study area, the plain along with the Xiangjiang river distribution on both sides. However, the resulting waste gas and waste due to the influence of human activities make the soil and water under the serious influence of heavy metal pollution. The irrigation of polluted water makes crops under heavy metal stress, which further affects human health. Previous studies have shown that Cd is the predominant pollutant in paddy soils that are watered from the Xiangjiang River, which contains industrial wastewater discharges [55,56]. The classification results in Figure 1d show that about 35% of the land is used for agricultural farming, 47% of the land is occupied by forests, and the rest is occupied by residential settlements, factories, rivers, and roads. The urban area is mainly distributed along rivers, mainly located in the northwest and central regions of the study area. Classification results were downloaded from the Geographical Information Monitoring Cloud Platform (http://www.dsac.cn).

Rice is the dominant crop in this region; for the single rice cropping region in Hunan Province, the rice is often transplanted in early June and harvested in late September. Consideration of the difference in heavy metal content in different regions, on the basis of field measurement and laboratory analysis, three experiment sites were selected based on the National Secondary Standard in the Environment Quality Standard for Soils (Figure 1c). The concentrations of heavy metals and nutrient substance in the soil samples were measured using an atomic absorption spectrophotometer at the Chinese Academy of Agricultural Sciences. The concentration of nutrient substances showed that the content of rice nutrient in the three experimental sites was inconsiderably different; hence, the growth of rice in the experimental areas was unaffected by nutrient stress. The average concentrations of the heavy metals Pb, Hg, and As in the samples were within acceptable environmental quality standards. However, the concentration of Cd in Site C was significantly greater than the upper limit value of the quality standards; the concentration of Cd in Site B was within the quality standards, and in Site A almost with no pollution. According to the concentration of Cd in soil, which was considered the main pollutant in the study area, Site A was categorized as “No pollution”, Site B was categorized as “Low pollution”, and Site C as “High pollution” (Table 1).

### 2.2. Field Measurements

Field measurements were designed to represent the rice variability within the study area. On the basis of the information provided by farmers about rice growth, rice of the same growing season was selected and reliable sampling was achieved to cover the difference in the study sites as much as possible. A series of measurements were taken in each sampling to characterize the accurate measurements, and these measurements were then averaged for each sampling. The measurements mainly included ASD data, Chl content, LAI, and geographic location. An ASD FieldSpec Pro spectrometer (Analytical Spectral Devices, Boulder, CO, USA), which can acquire a continuous spectral range of 350–2500 nm with sampling intervals of 2 nm, was used in canopy reflectance measurements. All spectra were collected under cloudless days between 10:00 and 14:00 at Beijing local time. The reflectance of a white BaSO4 panel calibration measured before every reflectance was taken. Then, the reflectance was calculated as the ratio between energy reflected by the canopy and energy incident on the canopy. Each canopy reflectance was the average of multiple scans automatically acquired by the FieldSpec. Chl was estimated using a SPAD-502 chlorophyll meter (Minolta Corporation, Ramsey, NJ, USA). LAI was estimated using a LICOR LAI-2000 Plant Canopy Analyzer (CID Bio-Science Inc., Camas, WA, USA), from which ALA could also be obtained. GPS was used to collect the geographic location of each sampling. Table 2 shows the variables measured in the field.

### 2.3. Sentinel-2 Satellite Imagery

The Sentinel-2 satellite imagery provides 13 spectral bands, ranging from visible and near-infrared to short-wave infrared, with four bands (B2–B4, B8) at 10 m spatial resolution, six bands (B5–B7, B8a, B11, B12) at 20 m spatial resolution, and three bands (B1, B9, B10) at 60 m spatial resolution [57]. Sentinel-2 satellite imagery has high quality of radiation and geometry. The processing level 1C product is geo-coded with subpixel accuracy and includes the top of atmosphere reflectance. The images collected in 2017 and 2018 were downloaded from the European Space Agency Sentinels Scientific Data Hub (https://scihub.copernicus.eu). SEN2COR tools (http://step.esa.int/main/third-party-plu-gins-2/sen2cor/) were used for atmosphere correction of the Sentinel-2 images. The spatial resolution was aggregated to 20 m resampling from B2 to B8A. All images were collected with minimal cloud cover in the study area, and the time was basically the same as that measured in the field [58]. An overview of the Sentinel-2 bands is presented in Table 3.

## 3. Methods

**Figure 2:**
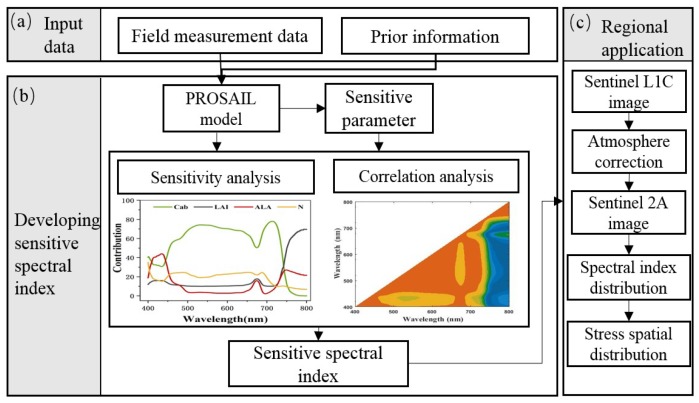
Flow chart of developing spectral index for detecting Cd stress in rice on a regional scale.

To develop a sensitive spectral index to detect the rice under Cd stress, the following methods were implemented (Figure 2): (1) Sensitivity analysis was conducted in the PROSAIL model to determine the most sensitive parameter to Cd stress. (2) The sensitive spectral index was developed based on sensitivity analysis and correlation analysis. (3) The sensitive spectral index was used to detect rice under Cd stress on the regional scale.

### 3.1. Rice Canopy Reflectance Simulations and Sensitivity Analysis

Spectral analysis was performed by using the radiative transfer model PROSAIL to analyze the effects of various biochemical and biophysical parameters of rice on the spectral reflectance of canopy under Cd stress and to obtain the parameters of subtle changes. The PROSAIL model is a canopy RTM radiative transfer model coupled by leaf (PROSPECT) and canopy radiative transfer model (SAIL) [39,59]. The PROSPECT model requires the following parameters: Chl, Cw, leaf dry matter content (Cm), carotenoid content (Car), and N. The simulated leaf spectrum can be used directly as an input to the SAIL model. The SAIL model requires LAI, ALA, hot spot parameter (S_L_), and solar and view geometries as parameters [39].

On the basis of the field measurement results of rice biochemical and biophysical parameters, the study parameters were set as follows: The Chl content was set in the range of 30–80 μg·cm^−2^, with a step of 0.3 μg·cm^−2^; the LAI value was set in the range of 1.5–6, with a step of 0.1; and the ALA value was set from 30 to 60°, with a step size of 1°. The values of these parameters were all over their plausible range according to the field measurements, and all measurements of each parameter were included [60].

Prior information as the model input parameter is critical for model simulation. Healthy leaves have values of N ranging from 1.5 to 2.5; the larger the values of N are, the more disordered the internal structure of the leaves will be [37]. Cd stress will disorder the internal structure of leaves, further increasing the value of N. When the value of N is greater than 2.5, the internal structure of leaves changes, and the vegetation is considered under Cd stress [15]. N inversion using the PROSAIL model, and combined with the inversion results and prior information, the values of N were set in the range of 1.5 to 3, considering the different levels of Cd stress [61,62]. Other input variables were set as a fixed reasonable value on the basis of field measurements and prior information [63,64]. Table 4 shows all the parameters.

A sensitivity analysis evaluates the relative importance of each input variable in a model and can be used to identify the most influential variables affecting model outputs [65,66]. Liang et al. [67] used sensitivity analysis to select the optimal vegetation index for LAI. Zhou et al. [43] selected the wavelengths which are sensitive to equivalent water thickness but insensitive to N and dry matter content by using sensitivity analysis. In this study, using global sensitivity analysis can not only reflect the sensitivity of each input parameter to the model simulation results, but can also comprehensively consider the influence of the interaction among the parameters on the model results. Hence, a global sensitivity analysis was conducted to identify wavelengths that parameters have a large influence on the spectral change under Cd stress. This approach was performed by analyzing the relative contribution of each input parameter to the variability of the output reflectance of the PROSAIL model. Sobol’s sensitivity analysis is a global sensitivity analysis method and was used in this study, which was performed using MATLAB software tool (GSAT) (MathWorks, Natick, MA, USA) [66,68,69]. For each input parameter, a list of wavelengths was derived based on their sensitivity.

### 3.2. Sensitive Biochemical and Biophysical Parameter Selection for Cd Stress in Rice

**Figure 3:**
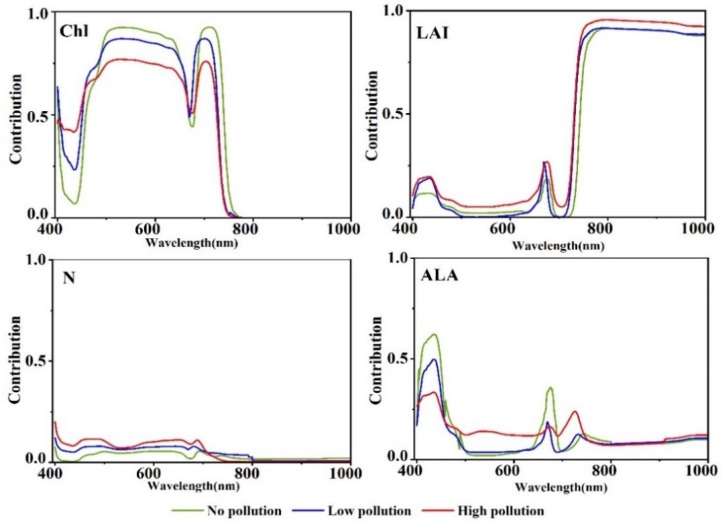
Global sensitivity analysis of four input parameters of the PROSAIL model for nonpolluted and polluted rice. Chl, chlorophyll; LAI, leaf area index; N, leaf structure parameter; ALA, average leaf angle.

In natural farmland ecosystems, the Cd content is relatively low, and the leaf morphology of rice and the internal cell structure of leaves are inconsiderably changed compared with leaves under high Cd content in laboratory environment [32,34]. In accordance with the field measurement results of the study area under different stress levels and the prior knowledge as the input parameters of the PROSAIL model, the sensitivity analysis results were obtained (Figure 3). The contribution of different parameters to canopy reflectance had its unique spectral contribution domain. In comparison with rice under no pollution, the sensitivity of Chl was remarkably decreased under Cd pollution; N, LAI, and ALA were changed but slightly. The change in Chl was about the sum of the changes in other parameters. Therefore, rice Chl was considered more sensitive to Cd stress than other parameters in environments with low Cd concentrations in farmland ecosystem.

### 3.3. Statistical Model

In order to understand the sensitive parameter and the applicability of the spectral indices under different stress levels, the Person correlation coefficient (*r*) was calculated to explore their correlation. The simple linear model was used to examine the relationships between spectral indices and biophysical and biochemical contents. The Pearson correlation coefficient (*r*) is calculated as:

(1) $$r=\frac{\sum_{i=1}^{n}\left(X_{i}-\bar{X}\right)\left(Y_{i}-\bar{Y}\right)}{\sqrt{\sum_{i=1}^{n}{\left(X_{i}-\bar{X}\right)}^{2}}\sqrt{\sum_{i=1}^{n}{\left(Y_{i}-\bar{Y}\right)}^{2}}}$$

where $X_{i}$ is the spectral indices value for samples, $\bar{X}$ is the mean of $X_{i}$, $Y_{i}$ is the biophysical and biochemical content, $\bar{Y}$ is the mean of $Y_{i}$, and n is the number of samples. The higher the absolute value of the correlation is, the stronger the linear relationships between the spectral indices and biophysical and biochemical contents are.

## 4. Result

### 4.1. New Spectral Index Development for Cd Stress in Rice

**Figure 4:**
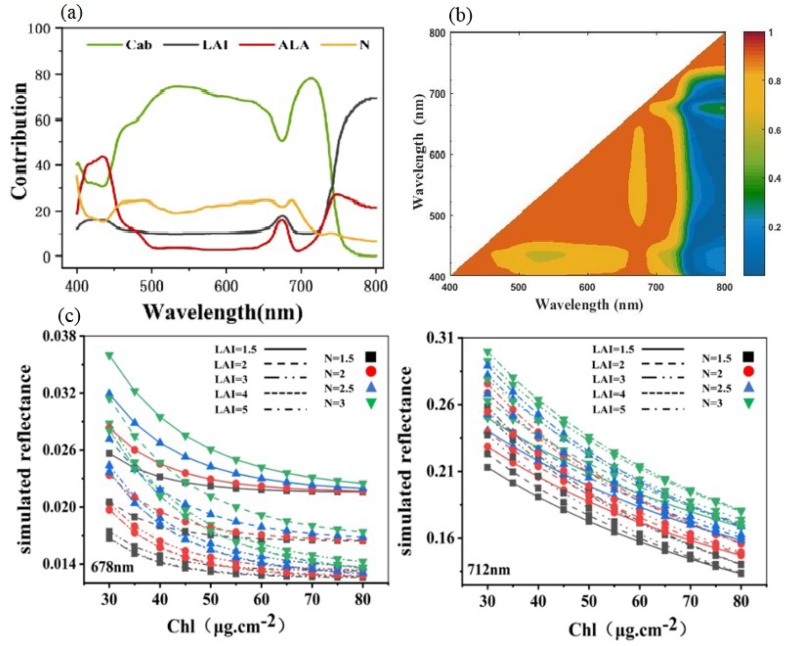
(**a**) First-order global sensitivity analysis results of four parameters in the range of 400–800 nm for PROSAIL model to reflectance; (**b**) PROSAIL-simulated reflectance from 400 to 800 nm coefficient of determination (R^2^) for all combinations of every two wavelengths (*n* = 1000); (**c**) Reflectance simulations with PROSAIL to assess the effects of LAI and N on Chl sensitivity wavelength.

From Figure 3, Chl is the most sensitive parameter for the spectral response of rice under Cd stress in the farmland ecosystem. This study explored Cd stress by developing a spectral index sensitive to Chl. The overall parameter range of each parameter was used as input, and the resulting sensitivity analysis is shown in Figure 4a. From the sensitivity analysis results, the main sensitive regions of chlorophyll were distributed in the spectral range of 480–720 nm, the sensitivity was 70–75%, and the sensitivity in the near-infrared wavelengths approached zero value. The sensitivity of LAI increased at 720 nm and was highly sensitive in the near-infrared region.

The correlation *R*^2^ between the two wavelengths in the wavelength range of 400–800 nm was calculated to obtain a combination of wavelengths with minimal redundant information. The two adjacent wavelengths were highly correlated, and the wavelength after 710 nm had a lower correlation with other wavelengths, except for adjacent wavelengths (Figure 4b). Chl had high sensitivity between 500 and 730 nm; depending on the correlation among wavelengths, 678 nm and 712 nm were finally selected as the optimal wavelengths. The 678 nm matched the absorption peak, and another sensitive wavelength of 712 nm was located in the “red-edge” region. Moreover, 550 nm was selected as the N-sensitive wavelength, and the sensitivity of LAI was high at 780 nm.

Further analysis of the simulated spectral reflectance of the PROSAIL model with parameter changes was performed to understand the influence of LAI and N on the sensitive wavelength of Chl. For the two Chl-sensitive wavelengths, the reflectance decreased with the increase in Chl content, and the relative change in reflectance at the red-edge position of 712 nm was significantly higher than 678 nm (Figure 4c). On the basis of this phenomenon and the sensitivity analysis result, the spectral index was considered the ratio of the reflectance in the red-edge to the reflectance in the absorption peak. This ratio result could effectively enhance the difference among different Chl levels. The higher the Chl levels are, the greater the value of the spectral index will be. Further, for the increase in LAI, the change in reflectance at 712 nm was more than that at 678 nm, and the difference between near-infrared and red edge reduced the effect of LAI on Chl. As N increased, the reflectance of the two wavelengths increased, and the Chl content was considerably affected at 678 nm. The ratio of reflectance at 678 nm and 550 nm was introduced to the spectral index to reduce the effect of N.

On the basis of the preceding results, the Chl-sensitive spectral index of rice was developed to reduce the interference of LAI and N on Chl and detect Cd stress accurately. The sensitivity spectral index heavy metal Cd stress-sensitive spectral index (HCSI) is defined as follows:

HCSI = (R780 − R712)/R678 × (R678/R550)(2)

### 4.2. Comparison Between HCSI and Common Spectral Index

The common spectral index was used to compare with HCSI to evaluate the reliability of HCSI in detecting rice Cd stress. A red-edge wavelength was used in HCSI. Four spectral indices based on red-edge wavelength were selected, namely, Clre, REP, the Moderate-resolution imaging spectrometer terrestrial chlorophyll index (MTCI), and NDRE. In previous studies, these indices have been indicated to be used to identify rice stress [70]. Table 5 presents the formulas for these spectral indices.

Table 6 shows the Pearson correlation coefficient (*r*) between spectral indices and Chl content simulated by the PROSAIL model, and between spectral indices and Chl content from field-measured data. A positive correlation was observed between the five spectral indices and Chl content. Comparison of the application effect of spectral indices in simulated and measured data showed that the correlation coefficient of HCSI was generally higher than other spectral indices under Cd stress. Comprehensive comparison of the changes in each spectral index under different pollution levels indicated that HCSI had an advantage when characterizing Cd stress over other indices.

### 4.3. Validation of HCSI for Detecting Rice Under Cd Stress

**Figure 5:**
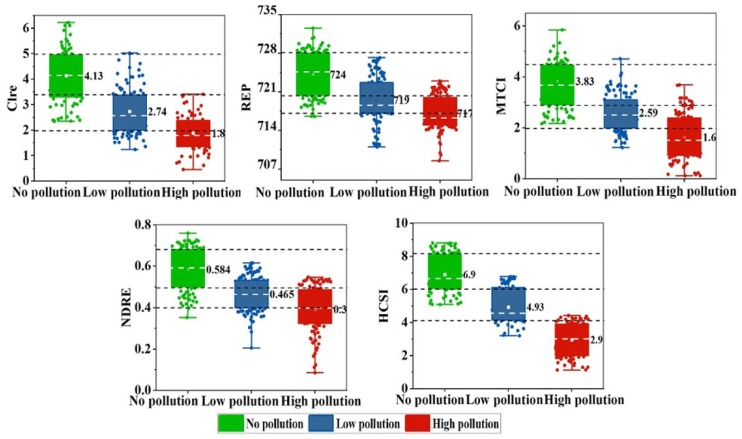
Five spectral indices in no pollution and pollution rice in different sites of field measurements in 2017.

**Figure 6:**
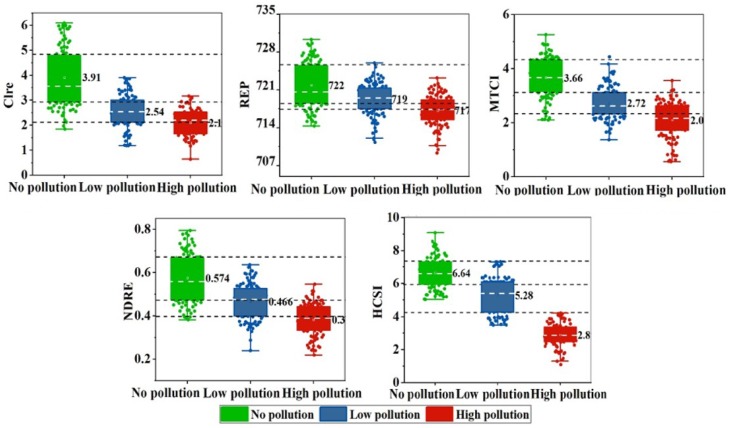
Five spectral indices in no pollution and pollution rice in different sites of field measurements in 2018.

The ASD field measurement data were used for validation to determine the capability of HCSI to identify stress for on-site measurement data. Five spectral indices were calculated for the rice ground points in the three experimental sites of 2017 and 2018. To describe the differences between the five spectral indices at three different stress levels, their data distribution under different Cd pollution levels is shown in Figure 4. The square symbol and horizontal line in the box represent the mean and median, respectively. The upper and lower horizontal lines denote the maximum and minimum values, respectively. The average ± standard deviation is the range of the box. The circles on the box are spectral indices data. From Figure 5, Clre, REP, MTCI, and NDRE all showed a larger overlapping range than HCSI in distinguishing different stress levels. In particular, HCSI performed better under the high level of stress. In 2017, the average HCSI value of Site A was 6.9, with a range of 5–9, which was two values higher than the average value of HCSI in Site B of low rice pollution and four values higher than the average value of HCSI in Site C of high rice pollution. The HCSI of low pollution ranged from 3 to 7, and the HCSI of high pollution was between 0 and 4. A similar difference was found in 2018 (Figure 6). The results indicate that the HCSI could be an effective indicator for detecting the stress level of Cd in rice in field measurements.

### 4.4. Regional Application of HCSI for Detecting Rice Under Cd Stress

The wavelength of the simulated canopy spectra generated by PROSAIL was 1 nm step. The simulated spectra previously mentioned were resampled into simulated Sentinel-2A spectra using the spectral response function of the Sentinel-2A bands to match the Sentinel-2A remote sensing image to the simulated data. With the Sentinel-2A spectra, the index was quantified using the following equation, in which the reflectance at the central wavelength of Sentinel-2A was used:

HCSI = (B07 − B05)/B04 × (B04/B03)(3)

**Figure 7:**
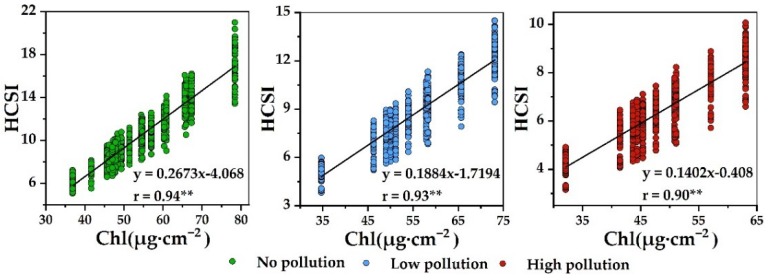
Relationships between Chl and HCSI using Sentinel-2A-simulated data in different heavy metal pollution levels (*n* = 1000).

The resampled Sentinel-2A band was adopted to construct HCSI and establish a correlation with Chl. The comprehensive analysis in Table 6 and Figure 7 shows that the spectral index established using the Sentinel-2A band was basically consistent with those established from the PROSAIL-simulated wavelength. Accordingly, the Sentinel-2A band could replace the spectral wavelengths simulated by PROSAIL to detect rice under Cd stress.

**Figure 8:**
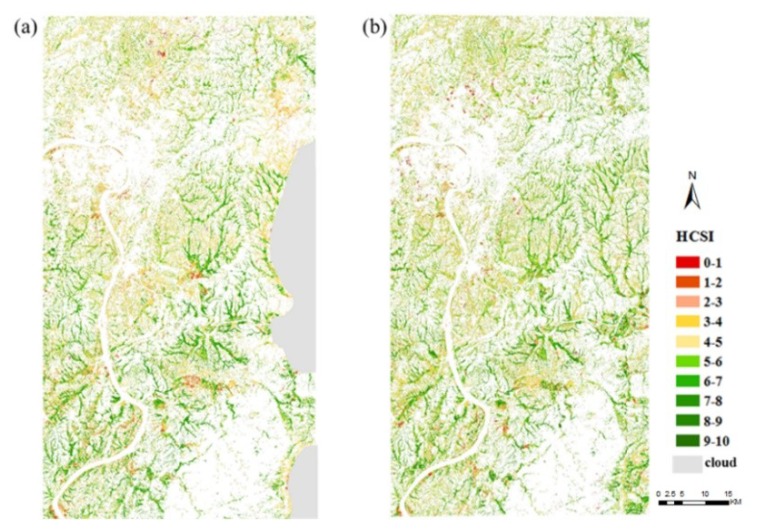
Spatial distribution of rice HCSI estimated from the Sentinel-2A remote sensing images in (**a**) 2017; (**b**) 2018.

**Figure 9:**
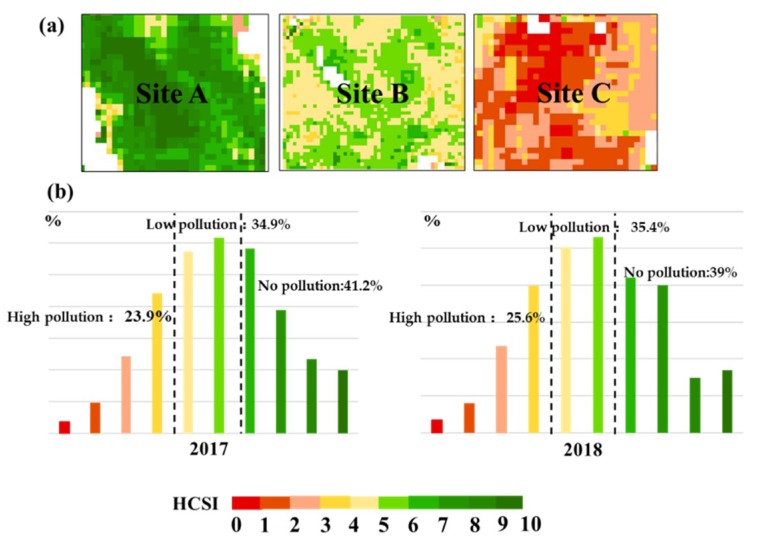
(**a**) Spatial distribution of HCSI in three field areas with different Cd pollution levels. (**b**) Histogram of HCSI into three categories for all rice areas in 2017 and 2018.

**Figure 10:**
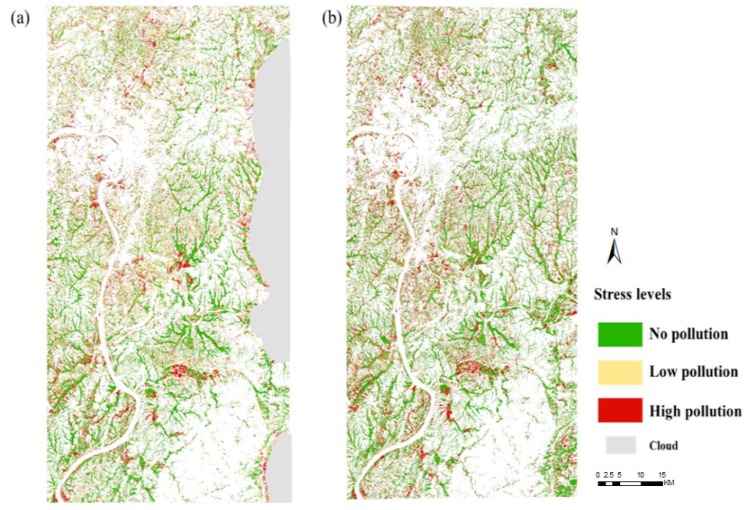
Spatial distribution of Cd pollution levels in rice: (**a**) 2017; (**b**) 2018.

The study extracted rice to avoid interference from other land types. Figure 8 shows the mapping results based on the 2017 and 2018 Sentinel-2A images established by HCSI. The mapping results were divided into 10 levels of color density. On the basis of the relationship between the HCSI values in Figure 9a and the pollution levels in the three experimental areas, the pollution levels in rice in Zhuzhou were categorized into three (Figure 9b). When HCSI ≤ 4, rice was under high pollution; when 4 < HCSI ≤ 6, rice was under mid pollution; and when HCSI > 6, rice was under no pollution. Figure 10 shows the regional spatial distribution of rice Cd stress levels in 2017 and 2018, with a similar spatial distribution of pollution levels over two years. The comparison of the classification result of the study area exhibited that the value of HCSI near the urban area was lower than that far away from the urban area, and the value of HCSI along the river was lower than that far away from the river. The areas where rice was under Cd stress were mainly distributed near the urban area in the northwest and central regions of the study area, which was near the river. Away from the urban area, the rice area near the forest was less or not affected by Cd stress. Figure 9b lists the detailed area statistical results of different stress levels in rice. Approximately 40% of rice was without Cd pollution, 35% was only polluted by low levels, and 25% of rice areas was polluted with high levels. The statistical results between the two years did not show considerable difference; the difference was only approximately 1%, which indicates that Cd stress did not obviously change in a short time.

## 5. Discussion

The main purpose of this study was to develop a new spectral index that can accurately detect different Cd stress levels in rice on a large scale. For that purpose, the radiative transfer model PROSAIL combined with measured data was used. The analysis found that chlorophyll was the most sensitive biochemical parameter to Cd stress in the farmland ecosystem. The new spectral index HCSI was developed based on chlorophyll. In comparison with the common spectral index, the HCSI can discriminate different stress levels more clearly. In addition, two years of Sentinel-2A remote sensing image data were used to predict the distribution of Cd stress, and the application of the new index has strong regional adaptability.

The satisfactory results obtained, on the one hand, differ from empirical approaches requiring several field measurement datasets for modeling to detect heavy metal stress [24,35,75], as using the vegetation radiative transfer model requires only a few field measurement biophysical and biochemical data to the range settings of the model parameters. In addition, because the vegetation radiative transfer model is generally applicable in different situations with good universality, the results can be easily applied to remote sensing data and, compared with research that generally uses the field-measured data or ASD spectral data for detecting heavy metal stress [27,32,76,77], it can be applied on a larger scale. On the other hand, the spectral characteristics and mechanism of rice under Cd stress in the natural farmland ecosystem were studied and analyzed, and the typical biophysical and biochemical characteristics of rice leaf and canopy scale were selected for discussion. The development of the index was to improve the ability to detect Cd stress by reducing the interference of other parameters for the sensitive parameter [78,79]. However, because of the complicated factors, a further investigation of the biophysical and biochemical parameter changes of rice under Cd stress needs more controlled strict experiments.

This study was carried out under the condition of known stress factor beforehand, and field collection was conducted on the nutritional status and heavy metal content status of rice, excluding the interference of some factors. Under a condition of known environmental Cd pollution, the Cd stress can be well detected by using HCSI. However, the impact on crops in the natural farmland ecosystem is complicated, and how to differentiate Cd stress from environmental stress, such as pest stress and temperature stress, is still a problem that needs further discussion. Two years of remote sensing data were used to predict the distribution of Cd stress; the spatial distribution of the pollution levels are similar in the two years’ image, and the area percentages of rice with the same degree of pollution in two years are similar, which indicates that rice under Cd stress is spatio-temporally stable. However, environmental factors are usually abrupt and do not last continuously [26,80]. Therefore, a study that combines spectral and temporal information and uses long time-series remote sensing data will help to distinguish the Cd stress from these environmental factors.

## 6. Conclusions

The PROSAIL radiative transfer model was used to develop a spectral index that can detect the subtle changes in rice under Cd stress in the natural farmland environment. The principal results and conclusions obtained can be summarized as follows: First, the analysis showed that chlorophyll is the most sensitive biochemical parameter to Cd stress in the farmland ecosystem, and the new spectral index HCSI developed based chlorophyll showed excellent performance compared with the common indices. Therefore, the proposed index can be considered a potential indicator for detecting Cd stress. Second, the application of HCSI to Sentinel-2A remote sensing images has strong applicability to detect Cd stress in rice at a regional scale. Furthermore, two years of Sentinel-2A remote sensing image data were used to predict the distribution of Cd stress, which confirmed that Cd stress in crops is characterized by stability in space and time. There is great potential to analyze and evaluate Cd stress on crops by using a radiative transfer model. In this study, the spectral index developed by the PROSAIL model was used to improve the universality and accuracy of detecting Cd stress in crops.

## Figures and Tables

**Table 1:** Average heavy metal concentration and nutrient substance in soil of three experimental sites.

Experimental Sites	Average Heavy Metal Concentration (mg/kg)				Nutrient Substance (g/kg)			Pollution Levels
	Cd	Hg	Pb	As	N	P	K	
Site A (27°43′40″ N, 113°14′24″ E)	0.09	0.15	35.15	11.64	2.79	0.771	4.88	No pollution
Site B (27°36′25″ N, 113°14′33″ E)	0.45	0.29	41.14	18.45	2.75	1.003	3.19	Low pollution
Site C (27°43′42″ N, 113°06′03″ E)	1.75	0.32	65.15	22.32	2.63	1.2	5.23	High pollution
Quality standard value	0.3–1.0	0.3–1.0	250–350	20–30				

Note: The quality standard value is the National Secondary Standard in the Environment Quality Standard for Soils (GB15618–1995).

**Table 2:** Field measurements of three biochemical and biophysical parameters used in PROSAIL model.

Parameters	Minimum	Maximum	Mean	Standard Deviation
Chl (μg·cm^−^^2^)	32.1	78.4	49.05	10.03
LAI (m^2^·m^−^^2^)	1.52	5.91	3.31	0.93
ALA (degrees)	37	57	43.5	5.68

Note: Chl = chlorophyll; LAI = leaf area index; ALA= average leaf angle.

**Table 3:** The overview of characteristics for Sentinel-2 bands after resampling and data collection.

Sentienl-2 Satellite Imagery			Data Acquisition	
Bands	Spatial Resolution (m)	Central Wavelength (nm)	Day/Month/Year	Percentage of Cloud
Band 2: Blue	10	490	12/07/2017	12%
Band 3: Green	10	560	24/07/2017	34%
Band 4: Red	10	665	37/07/2018	0
Band 5: Red-edge 1	20	705		
Band 6: Red-edge 2	20	740		
Band 7: Red-edge 3	20	783		
Band 8: Near Infrared (NIR)	10	842		
Band 8A: NIR narrow	20	865		

**Table 4:** Parameters used in simulating reflectance with PROSAIL model.

Model	Parameters	Symbol	Unit	Value
PROSPECT-5 (Leaf parameters)	Leaf parameter structure	N	-----	1.5–3 step:0.1
	Leaf chlorophyll content	Chl	μg·cm^−2^	30–80 step: 0.3
	Leaf carotenoid content	Car	μg·cm^−2^	0.0036
	Leaf dry matter content	Cm	g·cm^−2^	0.0064
SAIL (Canopy parameters)	Leaf water content	Cw	g·cm^−2^	0.005
	Leaf area index	LAI	m^2^·m^−2^	1.5–6 step:0.1
	Average leaf angle	ALA	degrees	30–60 step:1
	hot spot parameter	S_L_	-----	0.2
	Diffuse incoming solar radiation	SKYL	-----	25
	Solar zenith angle	θ_s_	degrees	30
	View zenith angle	θv	degrees	0
	Relative azimuth angle	ϕ_sv_	degrees	0

**Table 5:** Selection of four spectral indices related to red-edge wavelength in this study.

Spectral Index	General Formula	Reference
Red-edge chlorophyll index (Clre)	[R760 − R800]/[R690 − R720] − 1	Gitelson et al. [71]
Red-edge position (REP)	705 + 35(0.5(R665 + R783) − R705)/(R740 – R705)	Guyot and Baret [72]
Moderate-resolution imaging spectrometer terrestrial chlorophyll index (MTCI)	(R750 − R710)/(R710 − R680)	Dash and Curran [73]
Normalized red-edge differences (NDRE)	(R783 − R705)/(R783 + R705)	Barnes et al. [74]
Heavy metal Cd stress-sensitive spectral index (HCSI)	((R780 − R712)/R678)(R678/R550)	This study

**Table 6:** Pearson correlation coefficient (*r*) between spectral indices and Chl content (** *p* < 0.01).

		Clre	REP	NDRE	MTCI	HCSI
Simulated data (*n* = 1000)	No pollution	0.93 **	0.89 **	0.91 **	0.93 **	0.95 **
	Low pollution	0.89 **	0.88 **	0.88 **	0.90 **	0.91 **
	High pollution	0.85 **	0.85 **	0.84 **	0.86 **	0.89 **
Measured data	No pollution	0.89 **	0.88 **	0.89 **	0.87 **	0.92 **
	Low pollution	0.88 **	0.84 **	0.78 **	0.85 **	0.89 **
	High pollution	0.83 **	0.55 **	0.68 **	0.83 **	0.85 **

## Supplementary Files

Supplementary File 1
